# Supplementary material for: Assessing the extent and public health impact of bat predation by domestic animals using data from a rabies passive surveillance program
Source: PLOS Glob Public Health. 2022 May 11;2(5):e0000357. doi: 10.1371/journal.pgph.0000357 (PMC10021327; doi:10.1371/journal.pgph.0000357)
Supplement: Attachment — Submitted filename: PGPH-D-22-00039.Response.Reviewers.docx [file pgph.0000357.s002.docx]

Additional Editor Comments (if provided):

I appreciate the novel approach to observe public health impact of bat predation by domestic animals. It would be comprehensive to add few more tables or figures for better data visualization and statistical analysis. Looking forward to see the revised version.

Response: Thank you very much. We have now provided additional information in table 1. We have also revised the map in Figure 2 to allow for improved visualization using public health authorities instead of municipalities.

**Reviewer #1**

Reviewer #1: In this paper, the authors use existing data concerning encounters between wild bats and cats, dogs, and humans from the CFIA, to evaluate the comparative exposure risks from these groups. The authors’ overall assessment is that the greatest risk of rabies exposure comes from free-roaming outdoor cats, and that given this exposure risk, policies to reduce free-roaming of outdoor cats would be a valuable public health and conservation measure. This paper addresses the important and underappreciated role in rabies transmission that cats can have, given that public perception of rabies is largely canine related, and although of limited scope, contributes a Canadian case study to the body of evidence pointing towards the risks of outdoor cat depredation of bats. Whilst the data has its limitations, specifically concerning inevitable under-reporting of encounters, I find the paper to be clear and succinct, with a reasonable discussion of the data and its limits.

Overall, I support the publication of this interesting manuscript. Below are some points that the authors should consider in order to clarify the manuscript.

Major points:

In general, the paper would benefit from a clear breakdown of the data involved (in the form of a table or figure) that can be referred to throughout. I believe that all of the data used in the paper is available, but not necessarily in the most comprehensive format.

Response R1.1: We have now included a more detailed Table 1 that includes the submission data and also the rabies data, we hope that this table along with an improved Figure 1 and 2 provides sufficient information.

Line 153: ‘Overall, submitted bats had contact with cats for 41.5% of all cases (n=2599), while 8.7% of bats had contact with dogs (n=542) (Figures 1 & 2). Cases with no known human contact comprised 49.5% of the submissions (n=3097).’ This differs from the abstract which states on line 29 ‘… 49.5% had no animal exposure’. Please clarify the nature of this 49.5% of submissions, as at present I’m unclear as to where these samples originate.

Response R1.2: We have now clarified this in the methods in lines 116 – 120 which now state: “Samples were classified as associated with a dog, cat, livestock or another animal. Bats were classified in a single, ‘no domestic animal contact’ group if there was direct or indirect human contact or bats were submitted for confirmatory testing in wildlife disease surveillance programs.”

We also clarify in lines 170 – 171: “Cases with no domestic animal contact comprised 49.5% of the submissions (n=3097).”

This section of the results would also benefit from the 0.3% of other animals mentioned in the abstract, so that the full 100% of sample origins is apparent.

Response R1.3: Thank you, we now provide this information in results. Lines 168-170 now read “The remaining animal exposures involved goat (n= 1), horse (n=8), livestock (n=1), other non-cat or dog companion pet species (n=9) and raccoon (n=1).”

Minor points:

For the paragraph including the above points (beginning line 153) I find the order in which the data are presented somewhat disjointed, as the results jump between the contact exposure history of submitted samples, to the rabies results, then back to the exposure histories. I would recommend that the initial paragraph focus on exposure history, and the second (currently line 173) contains all of the rabies positives data.

Response R1.4: Thank you, we have structured the results section with the first paragraph being the exposure history with domestic species and the second paragraph discussing the rabies prevalence data.

Figure 1: If possible, this figure would benefit from the legend not being faded, so that the colours match those on the map. I would also appreciate the labelling of the map with the abbreviations for each province as in Table 1.

Response R1.5: We have now upgraded Figure 1 and now present the data at the public health region level to allow the reader greater contrast so spatial patterns can be discerned and the colour gradation is easily visualized.

This figure and the text on line 157 show differing proportions of cat-associated submissions, would the authors care to comment on the possible reasons for such variation? Do the proportion of indoor to outdoor cats differ between these regions? If this data is available, it might be interesting to compare.

Response R1.6:

There are differences in the data set on reporting lifestyle so we didn’t feel that we could do a direct comparison. For example for Alberta and BC, the outdoor cat data percentages were 41% and 33% respectively but both had 44 and 36% lifestyle reporting rate compared to Ontario with only 8%. We agree this is a really important point and we expanded on it in the discussion. Lines 294 to 304 now read:

“Although poorly known, free-roaming rates of owned cats in Canada are estimated to range from 40-70% [14]. Public attitudes towards free-roaming in cats are likely influenced by municipal by-laws, climate, socioeconomic status and public awareness of the negative feline welfare, zoonotic and conservation consequences associated with free-roaming. Spatial patterns present in this study suggest there is substantial geographic variation in the extent of free-roaming in cats, but the lack of consistent reporting in rabies submission reports precludes analysis. Data on the broad-and fine-scale dynamics of free-roaming cat populations is needed by public health professionals to be able enact community-specific interventions [61] in order to reduce the community health burden and financial costs associated with feline-associated zoonoses [16,62].”

Figure 2: A final bar at the top or bottom of this figure with the cumulative results for all bat species would be useful. Perhaps the proportion of each bat species that tested positive for rabies (not per exposure group) could also be incorporated in some way, for a visual representation of which bat species to have the highest rabies prevalence.

Response R1.7: We have now included a top summary bar in figure 2 and we have included species-specific rabies data in Table 1. Due to the large discrepancies in sample size from the different bat species being submitted leading to very large confidence intervals for the rabies prevalence estimates, we felt rabies numbers would be best represented in a table format, but would be happy to accommodate any representation that the reviewer feels would be more effective.

Line 174: ‘There were 21 rabies-positive bats submitted after interaction with an outdoor cat, with a calculated prevalence in this group of 4.1%’ – does this calculation use the aforementioned 552 outdoor cat submissions? Since this prevalence does not quite match the one presented in the paper, I assume that this id due to several of the outdoor cat encounter submissions being unsuitable for testing. As such, a breakdown of the data including exposure group, total suitable for testing, and positive tests would be useful (as mentioned above).

Response R1.8: For the prevalence calculations we only used samples for which a definitive test result was available which we clarify so lines 132-133 now read:

“Samples that were classified as unfit for rabies testing were removed (n = 392) from all statistical analyses.”

We also provide a breakdown of this uncertainty in lines 197-199 where we state:

“For the bats interacting with free-roaming cats, 7.4% (N= 41) of the samples were unfit for sampling, compared to 5.5% in the indoor (N=3) or unknown cats (N=109)”

And lines 199-203:

“Dog-associated bats had a rabies prevalence of 12.8% (95% CI: 9.9 – 16.1%) and an unfit sample submission rate of 10.7% (N=58). Rabies prevalence was 9.7% (95% CI: 8.7 – 10.9%) in submitted bats with no history of cat or dog association with an unfit sample submission rate of 5.8% (N=180).”

Line 193: Are the models found in Table 2 all of the models that were tested, or the top three models? Were any other combinations of fixed and random effects considered?

Response R1.9: We only tested these three models as we had very specific hypotheses and now state this directly in the lines 139-142: “We estimated the influence of domestic companion animal exposure on the probability of a rabies-positive bat, while controlling for season, bat species, year and province through comparing three potential models.”

Line 276: The authors cite that a predicted 20% of wildlife caught by free roaming cats is returned to owners, and thus available for submission for rabies testing. Assuming that this figure is accurate, could the authors speculate on the true risk of rabies exposure to outdoor cats?

Response R1.10:

We now address this in lines 327-330 which now read:

“Applied to the dataset in this study, although we would not expect the rabies prevalence to change, the much larger number of potential hunting events means that the cumulative rabies risk and conservation impacts of any free-roaming cats would be five times higher than what is presented here.”

Line 277: It may be pertinent to mention that although feral cats will have a far greater risk of interacting with rabid bats, and therefore are a large ecological concern, interactions with humans will be far less common than owned cats. Could also mention however, that owned free-roaming cats may interact with the more at-risk feral cats, even if their owners do not.

Response R1.11: Thank you, we have expanded on these crucial points in several sections:

Lines 283-285 now read: “Due to increased interactions with rabies reservoir species or interactions with feral cats, free-roaming owned cats have an amplified risk of becoming infected by rabies themselves [11,19,49,50], with bat predation being an additional route.”

Lines 333-337 now read: “Feral cat predation of bats is a significant conservation threat [17,18,41,68], and the direct rabies risk presented by feral cats is a recognized public health concern [52,53]. However, because feral cats, by definition, have reduced direct human interactions, feral cats would be expected to have a more limited role in indirect rabies transmission through bringing bats directly to humans.”

Very minor:

Line 76: ‘there are multiple terrestrial mammalian reservoirs for rabies, such as dogs, fox, skunk, raccoon, and bats’ – I would not consider bats to be terrestrial reservoirs.

Response R1.12: Thank you, typo is corrected.

Line 83 now reads: “There are multiple mammalian reservoirs for rabies, such as dogs, fox, skunk, raccoon and bats…..”

Line 192: I believe that the table description should read ‘degrees of freedom’ as supposed to ‘degree of freedoms’.

Response R1.13: Thank you, typo is corrected.

**Reviewer #2**

Reviewer #2: This paper presents an interesting examination of the extent and public health impact of bat predation by domestic animals using data from a rabies passive surveillance program. The authors found rabies prevalence was higher in bats found by dogs (12.8%) and humans (9.7%) followed by outdoor cats (4.1%) and indoor cats (1.9%). Although there was lower rabies prevalence in bats encountered by cats compared to dogs and humans, the much higher number of cat-bat interactions results in a greater overall rabies exposure risk to humans from free-roaming outdoor cats. In this case, the authors suggested that preventing free-roaming in cats is an underutilized public health recommendation. Overall, it is a novel and good study for which I have several major comments. Please find them below.

The manuscript would benefit from a clear articulation of the hypothesis that underlies the work. This should be followed up by specific predictions that can be tested using the data collected during the research. The statement of objectives in this Introduction section between line 86 to line 100 does not do an effective job of setting the stage for this research. It does not move the work out of a more descriptive context.

Response R2.1: Thank you for this suggestion, we have rewritten this paragraph to more succinctly state what our hypotheses and predictions were for this study and have made adjustments in the discussion as well to reflect this clearer articulation. We hope that these edits have improved clarity and also highlight the statistical tests that were done.

In this study, these results about the differences in the numbers of bats tested between provinces, between bat species and between domestic animals contacting with bats were mostly descriptive. It is possible to test these differences by some statistical analysis.

Response R2.2: We limited our statistical analyses to the rabies probability and elected to only provide descriptive statistics for the numbers of bats for several reasons. Firstly, although we controlled for province and year in the models, we did not specifically test the differences in the number of bats submitted because of the opportunistic nature of the bat submissions and therefore we felt that bat returns would be influenced by provincial population size, differences in the protocols, testing decisions of the local public health authority responsible for submission decisions and differences in bat population sizes. We needed to control for these factors in our analysis and did so by including variables like province and species as random effects but we feel that a more formal analysis with these factors as main objectives would require a different design. It would certainly be an interesting study but sufficiently involved to warrant an analysis on its own. Nevertheless, we still believe that the information is important here, even if descriptive.

Although the authors considered bat species as a random factor in generalized linear mixed models, every bat species may has different susceptibility to the rabies virus. In this case, it seems to be necessary to consider phylogenetic relationships of the 14 species of bats during the statistical analysis.

Response R2.3: We agree with the reviewer that each bat species will have different susceptibilities to rabies and that this also depends on the rabies virus variant. We have added more detail on the random variance associated with bat species in lines 212-214 and highlight this issue in the caveats (lines 314-322; lines provided below). Limited data exists for susceptibilities of bat species for different strains, but Davis et al (2013) did demonstrate that little brown bats (M. lucifugus) were susceptible to both heterologous and homologous strains. In addition, Jackson et al (2008) reported that a cluster of isolates designated E. fuscus more closely with virus variants associated with L. noctivagans and with Lasiurus spp, than with two other E. fuscus variants (1 and 2), though the E. fuscus variants would all be associated with E. fuscus as the reservoir. Therefore, we suggest that given this uncertainty, by modelling bat species as a random variable, we control for the random effect associated with bats due to both phylogenetic and ecological differences between bats without explicitly including their phylogenetic history as would be done with a more complicated phylogenetic mixed model. Bats are a diverse and complex order and how their phylogenetic relatedness affects susceptibility to rabies and other disease would be a fascinating area of future study.

In this study, the different domestic animals had the different rate of encountering rabies positive bats, but the risk of rabies infection in domestic animals is difficult to determine because of the following two reasons. On the one hand, data of rabies prevalence in different domestic animals was lacking. On the other hand, every domestic animal may has different susceptibility to the rabies virus. Thus, the factors should be considered and discussed in making intervention recommendation for public health.

Response R2.4: We have now added a caveat in to inform the reader of these susceptibilities and made suggestions on how this could alter recommendations”

Lines 314-322 now read:

“Second, we acknowledge that susceptibility to rabies infection varies among domestic and wild animals, including bat species, which is further dependent on the rabies virus variant, virus dosage load and the site of inoculation [52,58–61]. Although variability may alter patterns of direct rabies transmission from domestic species, existing data is insufficient to justify any reduced public health recommendations for feline vaccination and prudent avoidance of exposure. Furthermore, domestic species susceptibility does not remove the risk when free-roaming animals are bringing a rabies reservoir species into direct contact with humans.”

In Figure 1, latitude, longitude, north and scale should be marked.

Response R2.5: We have now included this information on the new map in Figure 1.
